# OPRA-RS: A Hearing-Aid Fitting Method Based on Automatic Speech Recognition and Random Search

**DOI:** 10.3389/fnins.2022.779048

**Published:** 2022-02-21

**Authors:** Libio Gonçalves Braz, Lionel Fontan, Julien Pinquier, Michael A. Stone, Christian Füllgrabe

**Affiliations:** ^1^IRIT, CNRS, Université Paul Sabatier, Toulouse, France; ^2^Archean LABS, Montauban, France; ^3^Manchester Centre for Audiology and Deafness, School of Health Sciences, University of Manchester, Manchester, United Kingdom; ^4^School of Sport, Exercise and Health Sciences, Loughborough University, Loughborough, United Kingdom

**Keywords:** random search (RS), automatic speech recognition (ASR), hearing aids (HAs), prescription rule, age-related hearing loss (ARHL), insertion gains, compression thresholds

## Abstract

Hearing-aid (HA) prescription rules (such as NAL-NL2, DSL-v5, and CAM2) are used by HA audiologists to define initial HA settings (e.g., insertion gains, IGs) for patients. This initial fitting is later individually adjusted for each patient to improve clinical outcomes in terms of speech intelligibility and listening comfort. During this fine-tuning stage, speech-intelligibility tests are often carried out with the patient to assess the benefits associated with different HA settings. As these tests tend to be time-consuming and performance on them depends on the patient's level of fatigue and familiarity with the test material, only a limited number of HA settings can be explored. Consequently, it is likely that a suboptimal fitting is used for the patient. Recent studies have shown that automatic speech recognition (ASR) can be used to predict the effects of IGs on speech intelligibility for patients with age-related hearing loss (ARHL). The aim of the present study was to extend this approach by optimizing, in addition to IGs, compression thresholds (CTs). However, increasing the number of parameters to be fitted increases exponentially the number of configurations to be assessed. To limit the number of HA settings to be tested, three random-search (RS) genetic algorithms were used. The resulting new HA fitting method, combining ASR and RS, is referred to as “objective prescription rule based on ASR and random search" (OPRA-RS). Optimal HA settings were computed for 12 audiograms, representing average and individual audiometric profiles typical for various levels of ARHL severity, and associated ASR performances were compared to those obtained with the settings recommended by CAM2. Each RS algorithm was run twice to assess its reliability. For all RS algorithms, ASR scores obtained with OPRA-RS were significantly higher than those associated with CAM2. Each RS algorithm converged on similar optimal HA settings across repetitions. However, significant differences were observed between RS algorithms in terms of maximum ASR performance and processing costs. These promising results open the way to the use of ASR and RS algorithms for the fine-tuning of HAs with potential speech-intelligibility benefits for the patient.

## 1. Introduction

The aim of hearing-aid (HA) prescription rules is to provide an appropriate level of amplification to restore audibility to hearing-impaired (HI) listeners while avoiding uncomfortable loudness levels. Established prescription rules—such as NAL-NL2 (Keidser et al., 2011), DSL-v5 (Scollie et al., 2005), and CAM2 (initially referred to as CAMEQ2-HF and commercialized in two software variants, CAM2A, and CAM2B; Moore et al., 2010b)—incorporate theoretical models of speech intelligibility and loudness perception. The amount of signal amplification is determined by frequency-specific insertion gains (IGs). As people with age-related hearing loss (ARHL) show a reduced dynamic range due to the elevation of hearing thresholds and loudness recruitment, compression of the signal amplitude is applied. When the input level exceeds a given threshold (referred to as the compression threshold, CT), the amount of amplification applied by the HA decreases more or less abruptly depending on the compression ratio (CR), defined as the increase in input level required for a 1-dB increase in output level. To determine IGs, in addition to the audiogram, prescription rules additionally take into account the number of HA channels, the maximum CR, the CTs, and the compression speed.

Initial HA fittings based on prescription rules generally lead to satisfactory clinical outcomes (Moore, 2008), but do not allow to address specific needs of the individual patient (Søgaard Jensen et al., 2019). As a consequence, audiologists often have to adjust HA settings for a given patient over several visits. During this fine-tuning stage, the benefits of HA settings are usually assessed using speech intelligibility tests. Since these tests can be lengthy, their administration may occupy a considerable part of the consultation. Also, the patient's performance can be affected by fatigue and loss of motivation (Sorin and Thouin-Daniel, 1983). Finally, given that speech intelligibility is influenced by the familiarity with the speech material (Hustad and Cahill, 2003) and that most speech intelligibility tests are composed of a fairly small set of items, the number of HA settings that can be assessed is limited.

In an effort to address these issues, Fontan et al. (2020b) demonstrated that, when combined with ARHL simulation, automatic speech recognition (ASR) can be used to assess the speech intelligibility benefits of specific IG functions in older HI patients listening through simulated HAs. An ASR system was used to quantify the benefits in speech intelligibility associated with IGs systematically varied relative to the CAM2 prescription by 0, ±3, or ±6 dB. Single-word recordings were amplified by an HA simulator, and then processed to simulate two of the perceptual consequences of ARHL based on the patient's audiogram, namely the elevation of hearing thresholds and loudness recruitment (Nejime and Moore, 1997). Finally, the recordings were fed to an ASR system to compute speech-identification scores. The IG function yielding the highest ASR performance and CAM2 gains were implemented in a simulated HA. Higher human speech-identification scores and subjective ratings of speech pleasantness were observed when speech was amplified with the ASR-based IG functions. The method used to determine these IG functions was named OPRA, an acronym for Objective Prescription Rule based on ASR.

To reduce processing costs, Fontan et al. (2020b) used only a large stepsize to vary IGs across a limited range and within four frequency bands, while keeping compression parameters fixed. These choices might have limited the observed amount of benefits associated with OPRA.

The aim of the present study was to extend previous work by using a broader range of possible gain values in five frequency bands, and a smaller stepsize for the variation of IGs. In addition, not only IGs, but also CTs were optimized. Given the number of parameters and possible values, a systematic assessment of all possible HA configurations, as done by Fontan et al. (2020b), would have been computationally extremely costly (in the present study, 7.77 × 10^18^ possible HA configurations would have to be assessed). The solution adopted in the present study was to use a random search (RS) approach, testing only a subset of all possible HA configurations. RS algorithms can be applied to a wide range of optimization problems, using different approaches, such as tabu search, ant-colony optimization, cross-entropy, multi-start and clustering, or genetic algorithms (for an overview, see Blum and Roli, 2001). While these approaches differ in their search procedure, they have in common to use probabilities for the exploration of the search space. The basic idea of the genetic RS algorithms used in this study is to vary randomly the value of several variables (here, IGs and CTs), and to quantify the result of this selection on the outcome variable (here, ASR performance). This process is repeated for *N* iterations. After each iteration, the new result is compared to the previous result. In case of an improvement, the search range is centered around the new configuration. From one iteration to the next, the search range is reduced by a constant factor. This results in the RS algorithm gradually converging on an optimal configuration. Three RS algorithms were tested: (i) one that tuned simultaneously CTs and IGs in all HA channels; (ii) one that tuned the CT and IGs for each HA channel, one after the other; and (iii) one that tuned in all HA channels first CTs, then IGs. As was done for OPRA (Fontan et al., 2020b), the current study exclusively focused on the optimization of speech intelligibility in quiet. It should however later be determined if the same method can be applied to speech in noise, and, in case of positive results, how it can interact with the signal-processing schemes currently developed to reduce the effects of background noise on speech intelligibility (for example, see the current Clarity challenge; Graetzer et al., 2021). More precisely, the current study aimed at addressing the following questions:

(i) As already observed for OPRA, does OPRA-RS yield higher ASR scores than CAM2?
(ii) Are there differences between the three RS algorithms in terms of ASR scores and speed (i.e., the number of iterations needed to reach a target ASR score)?
(iii) How reproducible are the outcomes of OPRA-RS, in terms of ASR scores, IG functions, and CTs?

## 2. Materials and Methods

### 2.1. Description of the OPRA-RS Processing Chain

**Figure 1:**
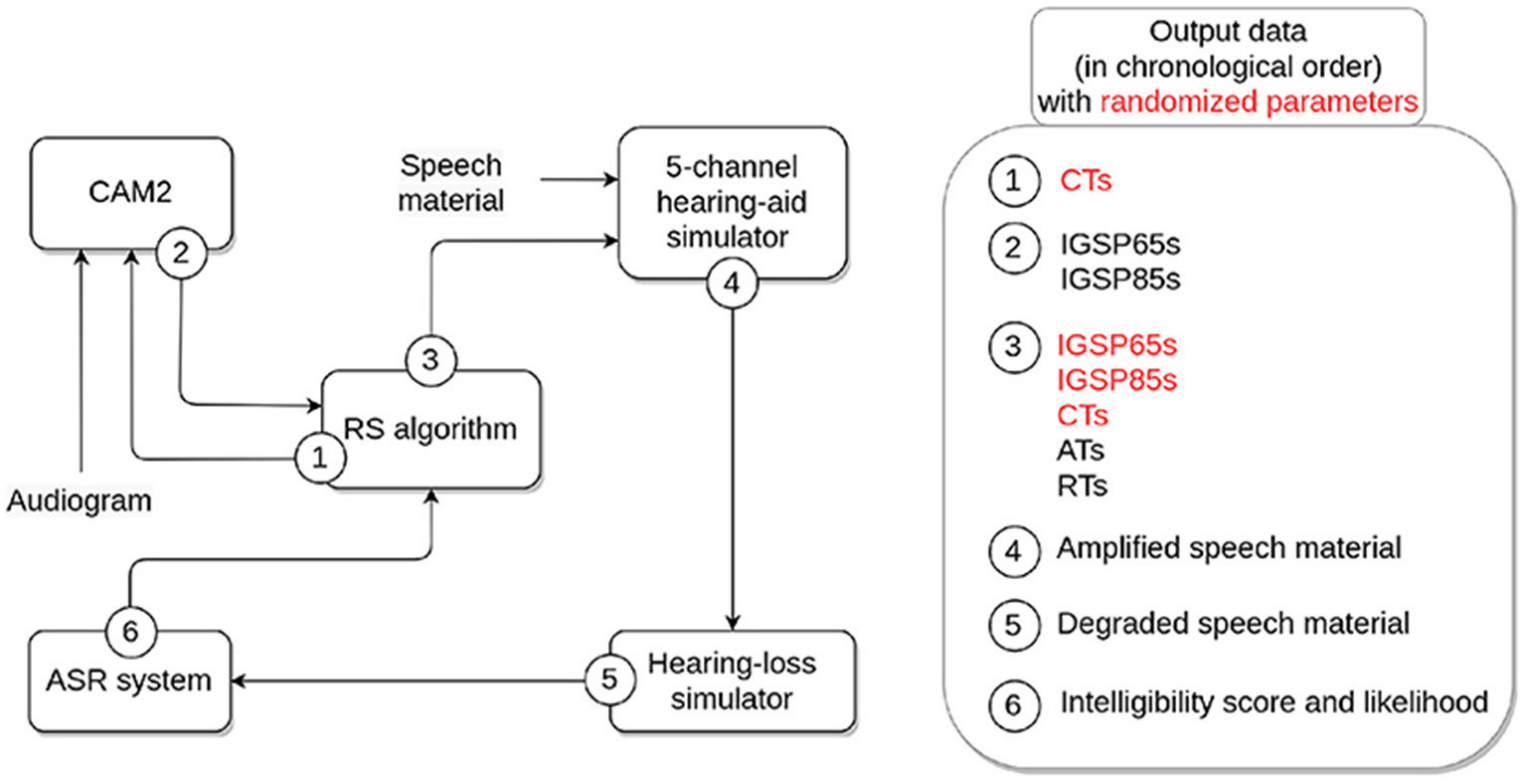
Overview of the components of the OPRA-RS processing chain and associated output data. HA parameters randomized by the RS algorithm appear in red.

Figure 1 details the different components of the processing chain used to generate the OPRA-RS-based HA settings for a given input audiogram.

First, the RS algorithm (implemented in Python; for a further description, see section 2.2) randomly defines CTs for each of the five frequency channels of the HA simulator used later in the processing chain. The audiometric thresholds and CTs are then inputted to the CAM2B-v2 software (Cambridge Enterprise, 2014) for the calculation of CAM2 IGs. This software also requires information about the frequency ranges of the HA channels (here, 0.1–0.7 kHz, 0.7–1.4 kHz, 1.4–2.8 kHz, 2.8–5.6 kHz, and 5.6—8 kHz) and the maximum CR allowed in the HA simulator (here, 10). In the present study, CAM2B-v2 was configured for an experienced HA user wearing a completely-in-the-canal HA, and assuming that the reference microphone for real-ear measurements was positioned near the tragus.

Second, IGs are defined by CAM2 for the two input levels for speech of 65 and 85 dB SPL (referred to as IGSP65 and IGSP85, respectively) at 11 center frequencies (0.125, 0.25, 0.5, 0.75, 1, 1.5, 2, 3, 4, 6, and 8 kHz), and fed back to the RS algorithm.

Third, the RS algorithm defines, for each of the parameters to be tuned, the range of values to be explored. Default search ranges were defined at the initialization of the algorithm (for more details, see section 2.3.2). At each iteration, the algorithm centers the ranges around the values that yield the best ASR performance, reduces the search ranges (by a constant factor), and assigns random values to each parameter within these ranges. For each channel, the algorithm then calculates the CR based on the IGSP65 and IGSP85 at the channel center frequency, following the equation:


(1) $$\begin{array}{r} \text{CR}=\frac{\Delta \text{input}}{\Delta \text{output}}=\frac{85-65}{85+\text{IGSP85}-\left(65+\text{IGSP65}\right)} \end{array}$$

Next, the algorithm checks that the CR falls within the range 1–10 (for more details, see section 2.3.2.3). If that is not the case, the following adjustments are applied until the CR falls within the desired range:

- If CR < 0, IGSP85s are increased by 0.5 dB;
- If 0 ≤ CR < 1, IGSP65s are increased by 0.5 dB;
- If CR >10, IGSP65s are decreased by 0.5 dB.

Then, the Speech Intelligibility Index (SII; American National Standards Institute, 1997) is calculated for the input audiogram and speech amplified according to (i) IGSP65s recommended by CAM2 and (ii) IGSP65s modified by OPRA-RS. The difference between the two SII values, (*SII*_CAM2_−*SII*_OPRA-RS_), is calculated. Depending on the sign of the difference, the current OPRA-RS IGs are either increased or decreased by 0.1 dB, and the difference in SII values is re-calculated. This process is repeated until the difference cannot be further reduced. This adjustment was implemented as the ASR system used by OPRA-RS normalizes the input signal, which means that its performance is not affected by changes in overall level. Only non-linear amplification (i.e., amplification that modifies the shape of the speech spectrum) impacts the ASR system's ability to recognize spoken words. As a consequence, using only ASR performance to guide the search for the best HA configuration might lead to a setting that would be inappropriate for actual patients in terms of audibility or loudness. The systematic SII-based adjustments of OPRA-RS IGs ensure that the audibility of the amplified speech is close to that of speech amplified according to CAM2, which aims at maximizing speech audibility while taking into account the overall loudness of processed speech (Moore et al., 2010b).

Fourth, an HA simulator (described in Moore et al., 2010a) is used to amplify 50 speech recordings of an adult male native-French speaker according to the HA settings received from the RS algorithm, for an input level of 65 dB SPL. Each recording consisted of the French definite article “le" followed by a disyllabic noun (e.g., “le parfum”—“the perfume.”) The speech material corresponded to five ten-word lists of the speech-intelligibility test developed by Fournier (1951), and which is commonly used in France for speech audiometry (Rembaud et al., 2017). The HA simulator, which is implemented in MATLAB^®^ (Mathworks, Natick, MA, USA), uses two dynamic range compressors implemented in series (the second one acting as a limiter; for more details, see Fontan et al., 2020b) in each of the five following frequency channels: 0.1–0.7 kHz, 0.7–1.4 kHz, 1.4–2.8 kHz, 2.8–5.6 kHz, and 5.6–8 kHz.

Fifth, the HL simulator, developed by Nejime and Moore (1997) and implemented in MATLAB^®^, is used to degrade the amplified speech material. The speech level at the output of the HA was used as the input level for the HL simulator. The simulator mimicks two of the perceptual consequences of ARHL: elevation of hearing thresholds (achieved by using linear filtering) and loudness recruitment (achieved by raising the signal envelope to a power; Moore and Glasberg, 1993). Loss of frequency selectivity was not simulated as it has been shown to decrease the strength of the correlation between ASR and human identification scores for speech in quiet (Fontan et al., 2020a).

Sixth, an ASR system is used to assess the intelligibility of the (amplified and degraded) speech material. The system is based on the ASR engine Julius 4.4.2 (Lee and Kawahara, 2009), and uses Gaussian Mixture Models (GMMs) and Hidden Markov Models (HMMs). Its acoustic models were trained using the Hidden Markov Model toolkit (HTK, version 3.4.1; Young, 1994) on the corpora ESTER (Galliano et al., 2006) and ESTER 2 (Galliano et al., 2009), consisting of approximately 100 h of recordings of radio broadcast news. The language model used by the ASR system is a finite state grammar designed to recognize the 50 noun phrases (i.e., article and noun) used in the study. Since the same article “le" is used in all noun phrases, ASR performance is calculated based on the recognition of final words (i.e., nouns). For each recording, the five words with the highest log-likelihood (a measure of the goodness-of-fit of the acoustic and language models to the speech signal) are returned by the ASR system. If the target word is included in the list, it is considered as recognized by the ASR system. Based on the processing of all recordings, two performance measures are computed: (i) the ASR score, which corresponds to the percentage of recognized words, and (ii) the average log-likelihood of all words that were recognized by the ASR system.

In summary, the input/output function of the whole OPRA-RS processing chain takes as its only input the audiogram. Three outcome HA settings are eventually provided by the system: the IGSP65s, IGSP85s, and CTs associated with the highest ASR performance.

### 2.2. Description of the Random-Search Algorithms

The present study used three genetic RS algorithms which are based on biologically-inspired operators, such as mutations (i.e., random variations) and selection (Goldberg, 1989). One algorithm simultaneously tuned CTs and IGs in all HA channels. For this algorithm, the number of possible HA configurations is very large, and this might compromise the convergence on optimal settings. Hence, two other algorithms were designed to reduce the number of possible HA configurations. The second algorithm simultaneously tuned the CT and IGs for one HA channel at a time, from channel 1 to channel 5. The final algorithm tuned, in all HA channels, first CTs and then IGs. Each of the three algorithms used a total of 750 iterations to optimize HA settings. Preliminary tests with the first algorithm showed that this number of iterations was sufficient to achieve near-ceiling ASR performance. The three algorithms, referred to as GEN1, GEN2, and GEN3, are further described in the following sections.

#### 2.2.1. Description of GEN1

**Figure 2:**
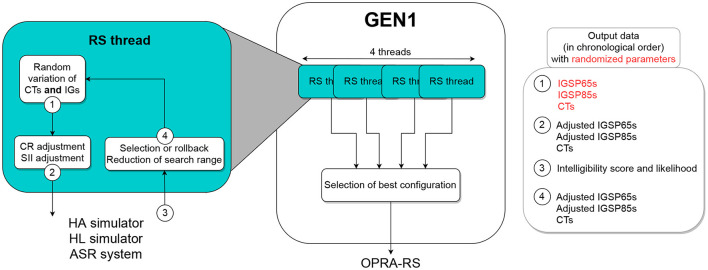
Schematic representation of GEN1, which simultaneously optimizes CTs and IGs in all HA channels.

The optimization process implemented in GEN1 is shown in Figure 2. In order to test a larger number of configurations, GEN1 is composed of four independent search threads, run in parallel. At initialization, each thread selects one HA configuration by assigning random values (picked from a uniform distribution) to all CTs and IGs. The IGs of this random configuration are then adjusted according to the constraints in terms of CR and SII. The configuration is sent to the other components of the processing chain (HA and HL simulators and ASR system in Figure 1). The ASR performance is returned to the RS thread and used together with the associated configuration as the baseline for the iterative search of the optimal configuration.

At each iteration *i* and for each parameter *P* to be tuned, a variation in the range from −Δ_*P*_ to Δ_*P*_ is randomly selected and applied to *P*. The value of Δ_*P*_ is initialized to half of the search range and decreased after each iteration following the equation:


(2) $$\begin{array}{r} \Delta_{P_{\text{i+1}}}=\Delta_{P_{i}}-\frac{\text{initial}\Delta_{P}-{\text{stepsize}}_{P}}{750} \end{array}$$

where stepsize_*P*_ corresponds to the step used to vary *P* (for details, see section 2.3.2). Following this equation, the final search range (i.e., the range explored during the 750th iteration) will equal stepsize_*P*_. The new configuration is then assessed. In the case where it yields a higher ASR score than the best configuration found so far, it becomes the new baseline for the application of random variations. Otherwise, a rollback is applied to return to the best configuration. In case that both configurations yield identical ASR scores, the log-likelihoods associated with the ASR scores are used to decide which configuration is retained. This process is repeated for 750 iterations in each of the four threads, and the best final configuration across threads is retained.

#### 2.2.2. Description of GEN2

**Figure 3:**
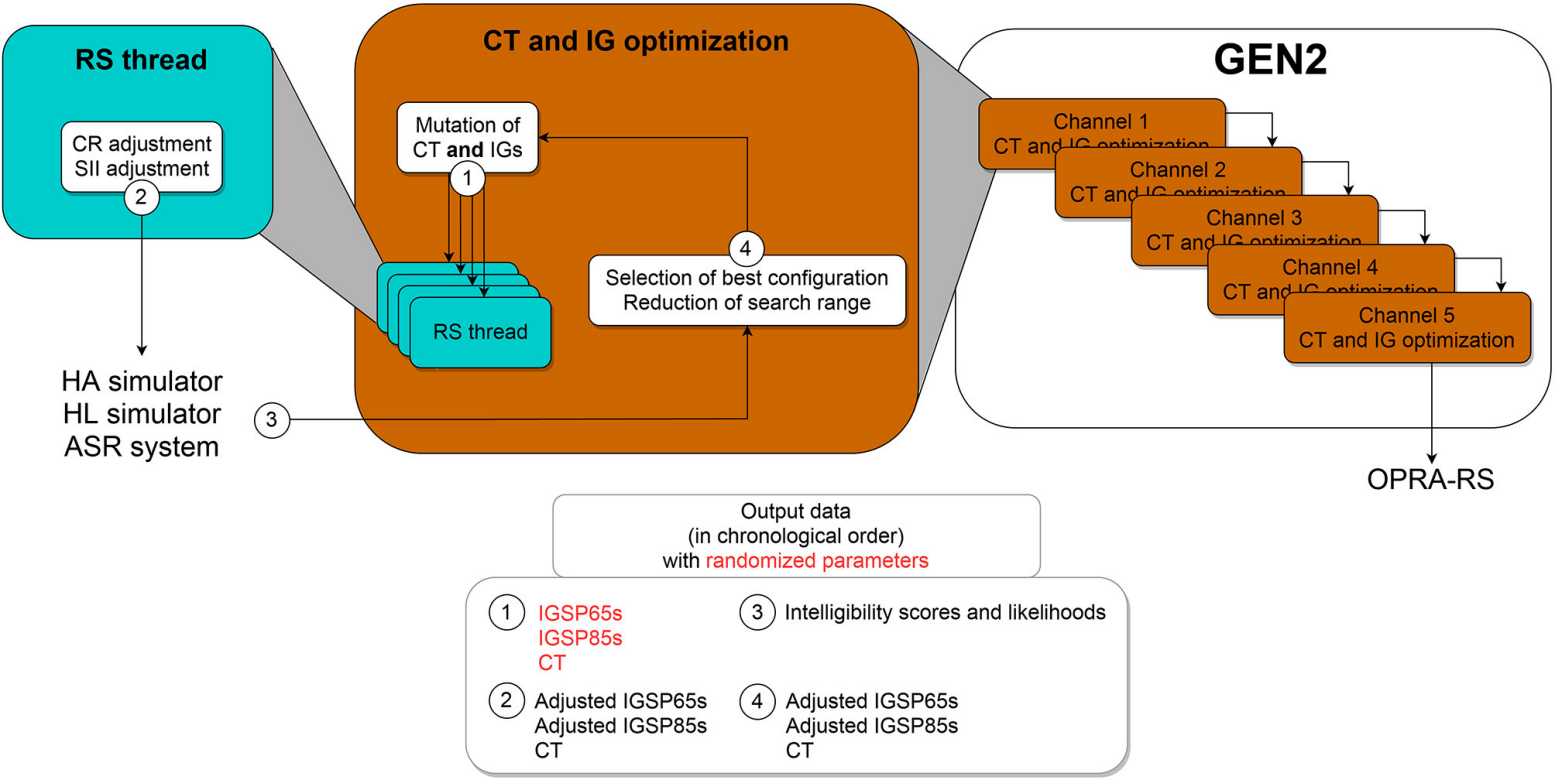
Schematic representation of GEN2, which simultaneously optimizes the CT and IGs for one HA channel at the time, one after the other.

In contrast to GEN1, GEN2 tunes the CT and IGs for each HA channel one after the other (Figure 3). For the optimization of CT and IGs in a given channel, four threads are run in parallel. At initialization, random values are assigned by each thread to all parameters and, following CR and SII adjustments, the resulting HA configuration is evaluated. Contrary to GEN1, GEN2 uses the best configuration found across all threads as the baseline for the next iteration.

At each iteration, the search range for each parameter is decreased around the best value (i.e., the value yielding highest ASR performance) found so far. This new search range is then [best value−Δ_*P*_*i*__; best value+Δ_*P*_*i*__] with:


(3) $$\begin{array}{r} \Delta_{P_{i}}=\left(750-i-1\right)\times \frac{{\text{half-range}}_{P}}{750} \end{array}$$

with half-range_*P*_ corresponding to the initial search range used for the parameter *P*, divided by two.

Once again, random variations are applied to all parameters within the target search ranges, and the resulting configurations are evaluated. In the case that the configuration yielding the highest ASR performance across all threads is better than the best configuration found up to the current iteration, it is used as a baseline for the next iteration. This process is repeated sequentially for 150 iterations in each of the five channels and the best final configuration across threads is selected.

#### 2.2.3. Description of GEN3

**Figure 4:**
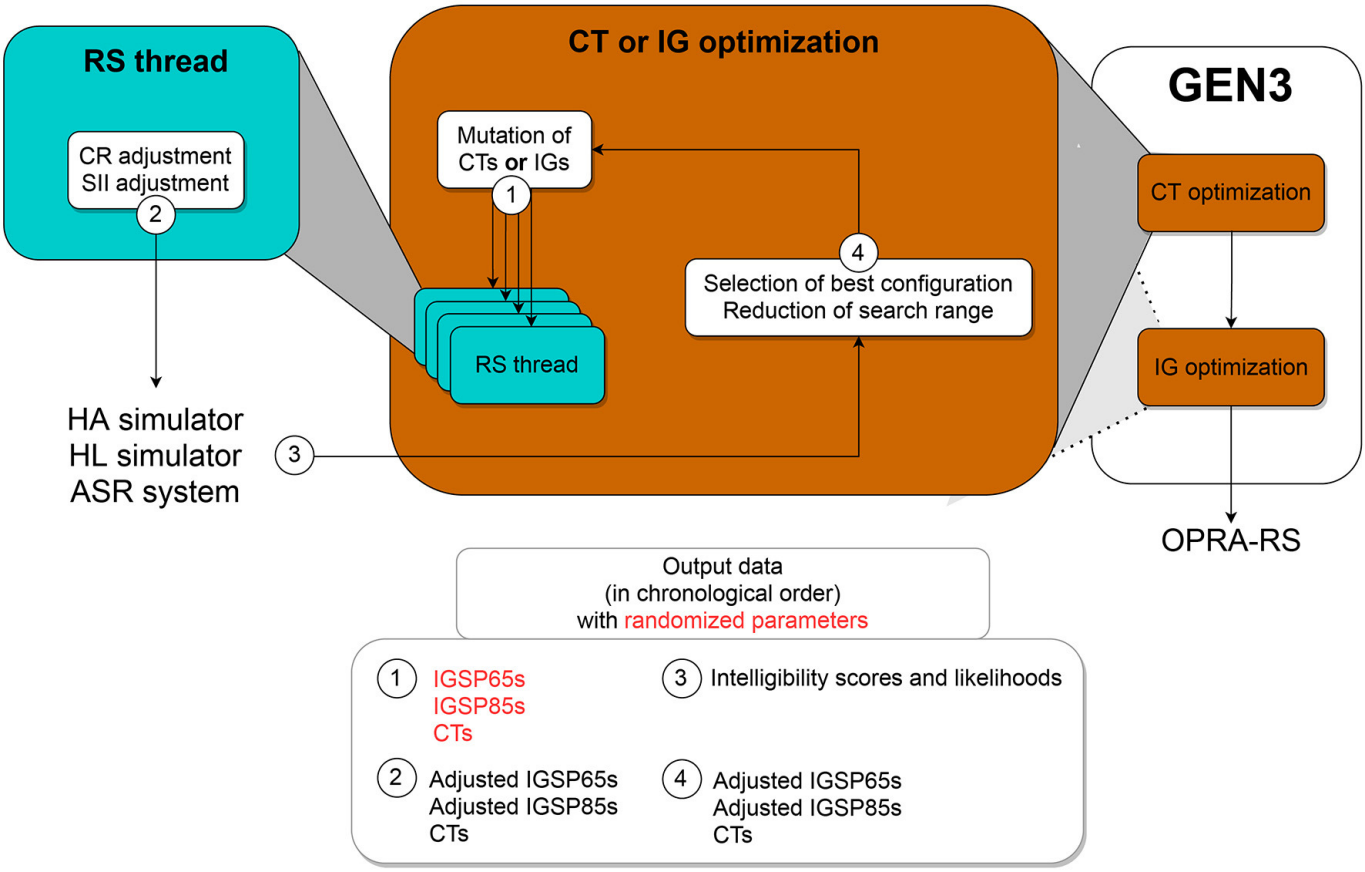
Schematic representation of GEN3, which optimizes first CTs and then IGs in all HA channels.

Similar to GEN1, GEN3 tunes parameters in all channels at the same time. However, GEN3 tunes CTs and IGs sequentially (first CTs in all channels, then IGs in all channels; see Figure 4).

Four threads are used for the search. As for the initialization of GEN1 and GEN2, at start each thread of GEN3 assigns random values to all parameters. After adjusting the IGs of this random configuration according to the constraints in terms of CR and SII, the configuration is evaluated. If it yields better ASR performance than the best configuration found so far, then it is retained and used by all threads as the new baseline for the search. Otherwise, a rollback is applied. After each iteration, the search range is reduced using the same equation than in GEN2 (see Equation (3)). This process is repeated 250 times for the tuning of CTs, and then 500 times for the tuning of IGs.

### 2.3. Experiment Protocol

#### 2.3.1. Input Audiograms

**Figure 5:**
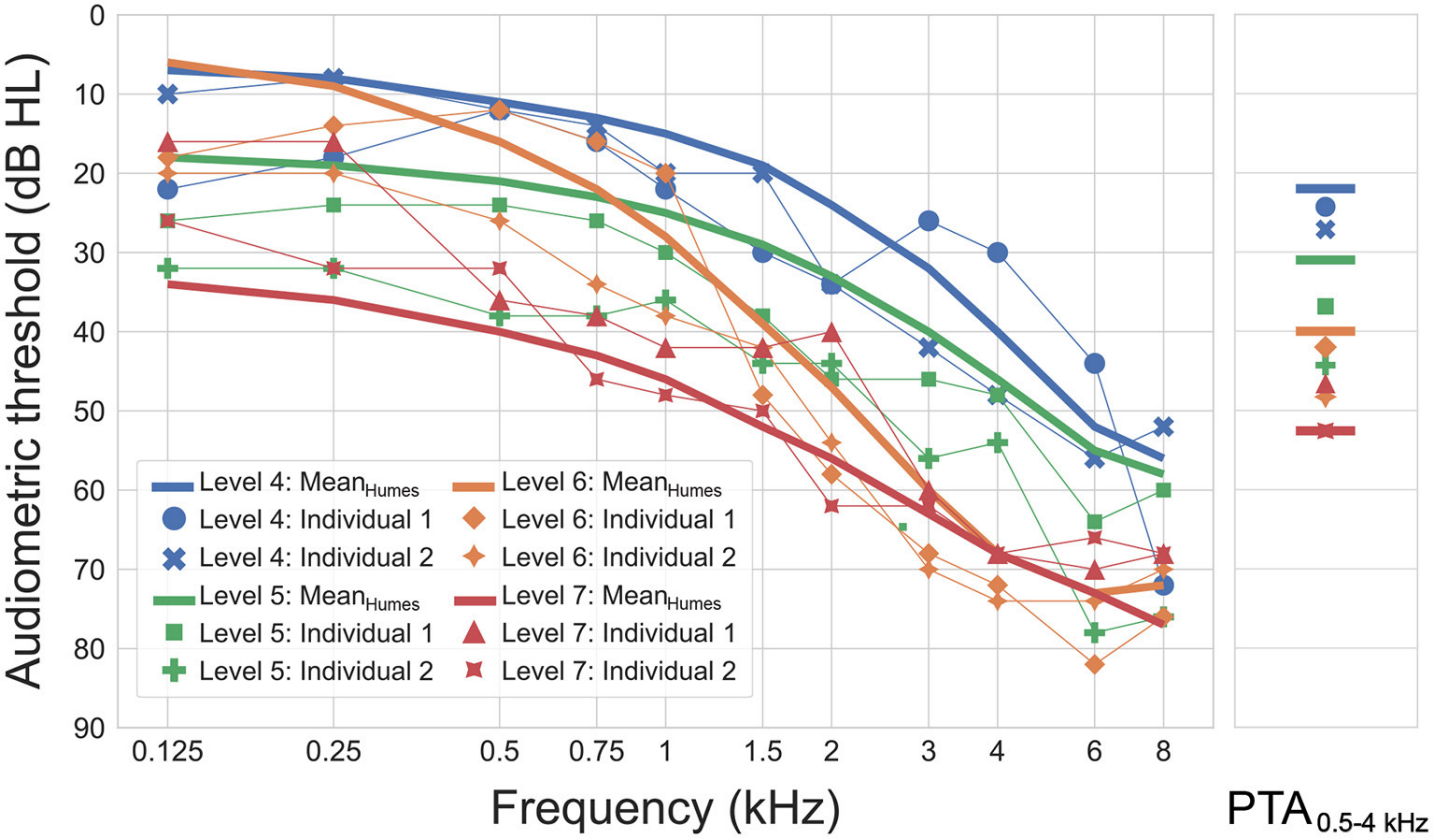
Individual and mean audiograms for different WARHICS levels used as input to the OPRA-RS processing chain (left panel). Pure-tone average (PTA) for frequencies between 0.5 to 4 kHz are shown for each audiogram in the right panel.

Figure 5 shows the four mean and eight individual audiograms that were used in the study. The mean audiograms correspond to the audiometric data reported by Humes (2021) for levels 4 to 7 of the Wisconsin Age-Related Hearing Impairment Classification Scale (WARHICS; Cruickshanks et al., 2020). As Humes (2021) did not report all hearing thresholds required for the HA and HL simulations, the missing values at frequencies of 0.125, 0.25, 0.75, and 1.5 kHz were intra- or extrapolated using 3rd-order least-squares polynomial fits.

The eight individual audiograms were selected based on them complying with the maximum frequency-specific threshold values defined by the WARHICS scale for levels 4 to 7. Two audiograms were selected for each of the four WARHICS levels. The individual audiograms came from eight older patients diagnosed with sensorineural HL (mean age: 70 years; age range: 63–78 years). Audiograms corresponding to different levels of HL severity were used in order to verify that OPRA-RS works for a wide range of HLs. Indeed, Fontan et al., 2020a observed that ASR performance is reduced for simulated severe-to-profound HLs. This might impact the search for the optimal HA configuration.

As can be observed in Figure 5, the mean audiograms show monotonic decline with increasing frequency, as is typical of ARHL. The individual audiograms follow the same general tendency, but are more erratic, which could impact the performance and outcome of the RS. The pure-tone average (PTA) of all individual audiograms bar one is identical to, or higher than the mean audiogram corresponding to the same WARHICS level.

#### 2.3.2. Random-Search Ranges and Variation Stepsizes

##### 2.3.2.1. Compression Thresholds

For the variation of CTs, 1-dB steps were used, and the lower and upper limits of the search range were 20 and 50 dB SPL, respectively. These choices were based on values used in previous studies implementing HA settings recommended by CAM2 in the same HA simulator as used here (Moore et al., 2010a, 2011; Moore and Sek, 2016).

##### 2.3.2.2. Insertion Gains

A step size of 0.1 dB was used to vary IGs. The lower and upper limits for OPRA-RS IGs were set to ±10 dB relative to the IGs prescribed by CAM2. This choice was based on previous studies that compared initial prescriptions to final HA settings (i.e., after fine-tuning based on self-adjusted user preferences). Søgaard Jensen et al. (2019) reported that IGs after fine-tuning differed from initially applied IGs by ±9.6 dB. Similar differences (of ±10 dB) were observed by Boothroyd and Mackersie (2017).

##### 2.3.2.3. Compression Speed and Maximum Compression Ratio

For people with severe HLs, it may be theoretically useful to use high CRs (>3) to restore audibility at a comfortable level (Moore, 2008; Moore and Sek, 2016). In the case of fast compression, such high CRs can lead to a loss of intelligibility due to distortions of the signal envelope (Verschuure et al., 1996; Souza, 2002). As this study included audiograms corresponding to mild-to-moderately-severe HLs, only slow compression speeds were used, with CRs allowed to vary from 1 to 10, as done by Moore and Sek (2016). More precisely, for the tuning of CTs and IGs, attack times (ATs) were set to 200, 100, 100, 100, and 100 ms, and release times (RTs) to 2000, 1500, 1200, 1000, and 1000 ms, for HA channels 1 to 5, respectively.

#### 2.3.3. Procedure

The OPRA-RS processing chain was run on the OSIRIM platform (http://osirim.irit.fr/site/en), a cluster of 928 central processing units and 28 graphical processing units. Six Intel^®^ Xeon^®^ Gold 6136 processors were used for the computation of OPRA-RS settings. Each RS algorithm was run twice to optimize CTs and IGs for each of the 12 input audiograms. A single run of any of the algorithms required an average processing time of 38 h.

#### 2.3.4. Statistical Analyses

The significance of overall differences in data distribution were investigated through linear mixed models, followed by paired comparisons. T-tests were used, except when the assumptions for parametric tests were not met, in which case Wilcoxon tests were used. In case of multiple comparisons, the Holm-Bonferroni correction was applied. To investigate the association between PTA and ASR scores or convergence speed, Spearman correlations were used since data were not normally distributed. Data from the two repetitions of the RS algorithms were used to assess the reproducibility of OPRA-RS outcomes; for all other analyses, only data from the first repetition were used. Only IGs specified for a 65-dB-SPL speech input level were used, as this was the level set in the HA simulator. For these analyses R (R Core Team, 2021), with *lmerTest* and *emmeans* packages, and SPSS Statistics version 23 (IBM, Chicago, IL), were used.

## 3. Results

### 3.1. Comparison of OPRA-RS and CAM2

**Figure 6:**
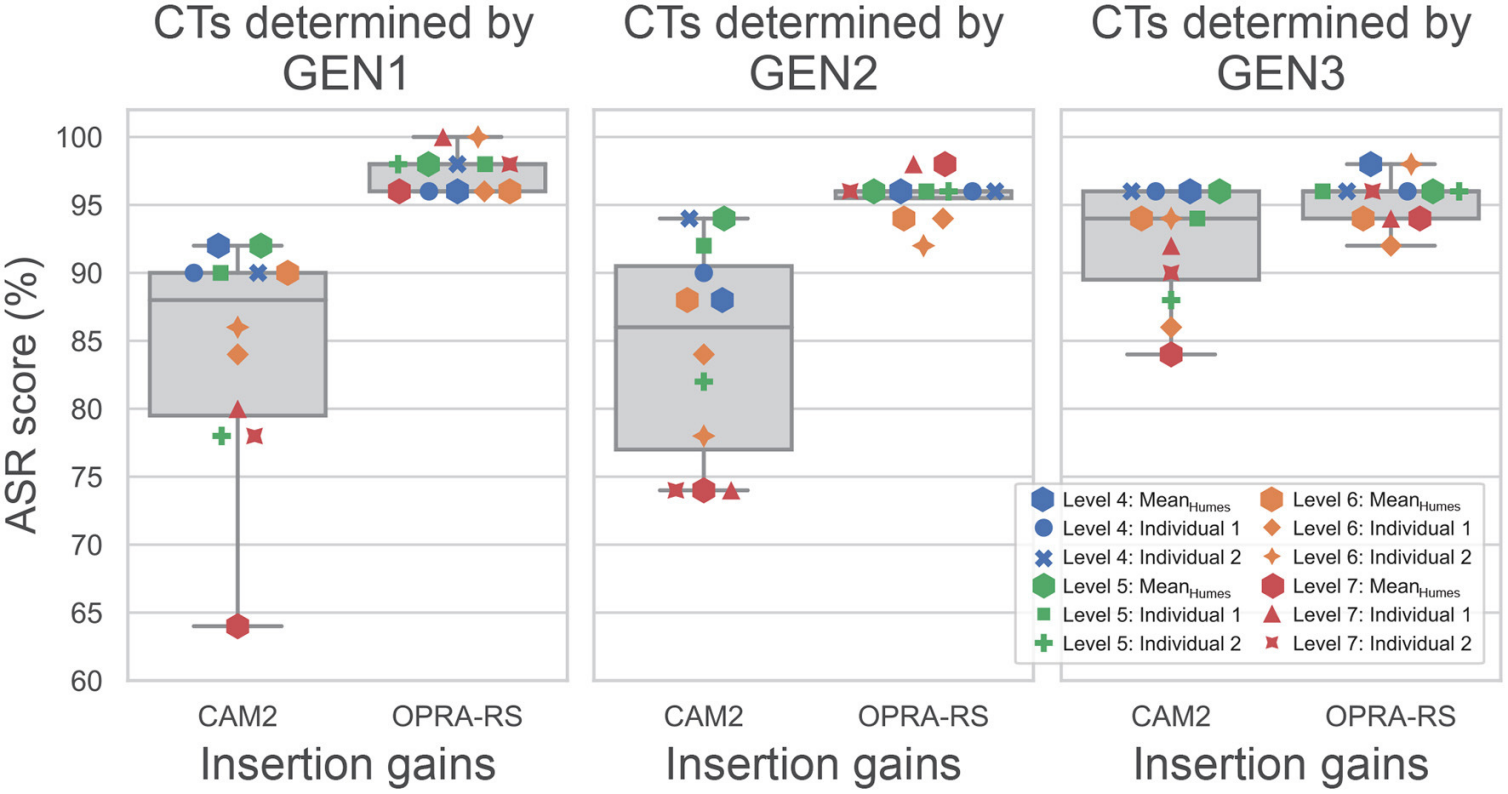
Distribution of ASR scores based on IGs selected by OPRA-RS or recommended by CAM2, for each of the three RS algorithms. The horizontal lines inside the boxes represent median ASR scores. Whiskers and horizontal limits of the boxes represent, from bottom to top, the 0, 25th, 75th, and 100th percentiles. In the case of OPRA-RS, the medians and 75th percentiles overlap.

To compare the ASR scores associated with OPRA-RS and CAM2, the optimal CTs selected by OPRA-RS algorithms were inputted to CAM2Bv2, as this software does not provide CTs. Figure 6 shows the distribution of ASR scores for the 12 audiograms with the IGs selected by OPRA-RS or recommended by CAM2. In all conditions, OPRA-RS prescriptions yielded ASR scores in excess of 90%, with median values of 98% for GEN1 and 96% for GEN2 and GEN3. By comparison, ASR scores associated with CAM2 prescriptions are more broadly distributed, and their median values are lower (88% for GEN1, 86% for GEN2, and 94% for GEN3). Wilcoxon signed-rank tests show that the differences between ASR scores obtained with the two prescription rules (10 percentage points for GEN1 and GEN2, and 2 percentage points for GEN3) are statistically significant (for GEN1: *Z* = 3.07;*p* = 0.002; for GEN2: *Z* = 3.06;*p* = 0.002; for GEN3: *Z* = 2.54;*p* = 0.011).

**Figure 7:**
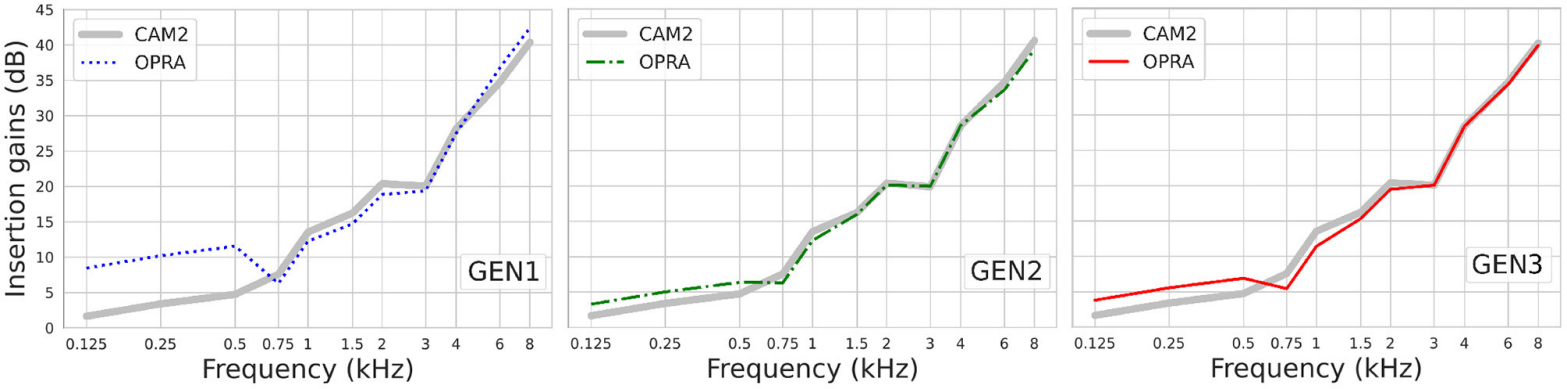
IG functions prescribed by OPRA-RS and CAM2, averaged across audiograms.

As the same CTs were used by OPRA-RS and CAM2, the observed differences in ASR scores are due to the IGs prescribed by the two rules. Figure 7 shows the IG functions averaged across audiograms, for OPRA-RS and CAM2, as a function of the type of RS algorithm. The IG functions for the two prescription rules are very similar (mean absolute difference of 1.7 dB), except for GEN1 for which, consistent with Fontan et al. (2020b), OPRA-RS tended to prescribe higher IGs (average absolute difference of 6.8 dB) for the low frequencies (0.125–0.5 kHz).

### 3.2. Influence of RS Algorithm Type, HL Severity, and Audiogram Type on CAM2

Differences between ASR scores associated with CAM2 can be observed across the three RS algorithms, with ASR scores higher for GEN3 than for GEN1 and GEN2. The statistical significance of these differences was assessed by a linear mixed model with the type of RS algorithm as a fixed effect and the input audiogram as a random effect. The results show that the type of RS algorithm is highly significant (*F*(2, 22) = 16.7;*p* < 0.001). Corrected *post-hoc* comparisons indicate highly significant differences between GEN1 and GEN3 (*t*(22) = −5.0;*p* < 0.001), as well as between GEN2 and GEN3 (*t*(22) = −5.1;*p* < 0.001). The difference between GEN1 and GEN2 is not significant (*t*(22) = 0.1;*p* = 0.915).

The lowest ASR scores are obtained for audiograms corresponding to level 7 of the WARHICS scale (i.e., the most severe HLs), whereas the highest ASR scores are obtained for audiograms corresponding to WARHICS levels 4 and 5. This suggests a negative association between HL severity and ASR scores obtained with CAM2. To assess the statistical significance of this relationship, Spearman's correlation was computed between ASR scores and the pure-tone average (PTA) for frequencies 0.5, 0.75, 1, 1.5, 2, 3, and 4 kHz. For all three RS algorithms, significant strong negative correlations are found (for GEN1: ρ = −0.88;*p* < 0.001; for GEN2: ρ = −0.85;*p* < 0.001; for GEN3: ρ = −0.82;*p* = 0.001).

Finally, ASR scores do not seem to be affected by the more erratic nature of individual audiograms. The distribution of ASR scores for mean and individual audiograms is rather similar, with lowest and highest scores observed in both cases.

### 3.3. Influence of RS Algorithm Type, HL Severity, and Audiogram Type on OPRA-RS

Only small differences are observed between ASR scores associated with OPRA-RS across the RS algorithms. Highest scores are found for GEN1 and only slightly lower scores were found for GEN2 and GEN3. The statistical significance of these differences was assessed using a linear mixed model with the type of RS algorithm as a fixed effect and the audiogram as a random effect. The results show that the type of RS algorithm has a significant effect on the ASR scores (*F*(2, 22) = 5.6;*p* = 0.011). Corrected *post-hoc* comparisons indicate significant differences between GEN1 and GEN2 (*t*(22) = 2.8;*p* = 0.023) and between GEN1 and GEN3 (*t*(22) = 3.0;*p* = 0.020). The difference between GEN2 and GEN3 is not significant (*t*(22) = 0.3;*p* = 0.81).

**Figure 8:**
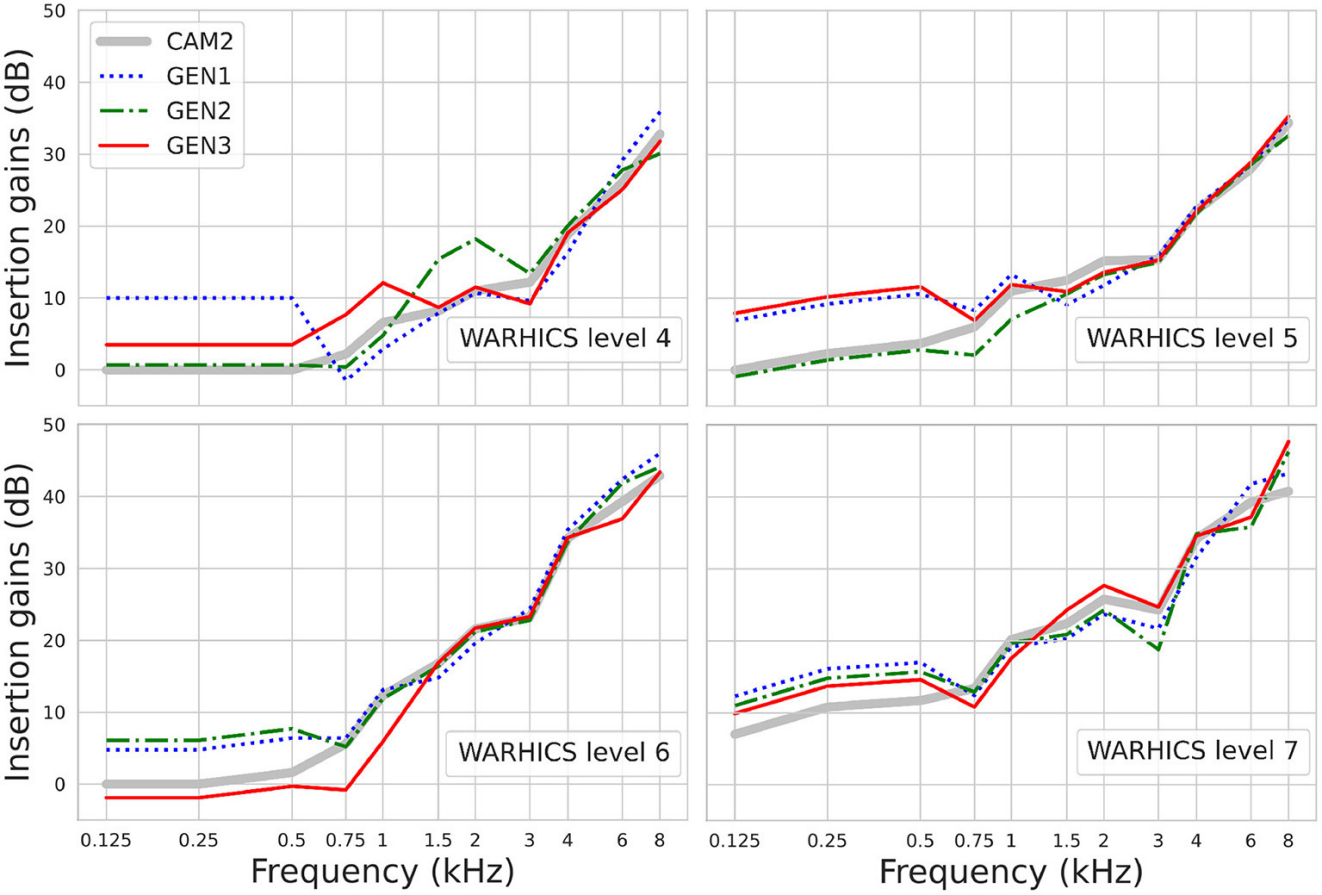
Insertion gains prescribed by GEN1, GEN2, GEN3, and CAM2 using Humes (2021)'s average audiograms corresponding to WARHICS levels 4 to 7.

Figure 8 shows the IGs prescribed by the three RS algorithms for Humes (2021)'s average audiograms corresponding to WARHICS levels 4 to 7. The largest difference observed between the IGs selected by the three RS algorithms is 9.3 dB. IG differences are often larger at low frequencies than at higher frequencies (see for example the IGs selected by GEN1 and GEN2 for the mean audiogram corresponding to WARHICS level 4). The results show that highest gains are not systematically prescribed by the same RS algorithm.

Spearman correlations do not indicate any association between ASR scores and PTA: for all three RS algorithms, correlation coefficients are weak (all ρ ≤ 0.35) and non-significant (all *p*≥0.28).

As for CAM2, the distribution of ASR scores suggests that the more erratic nature of individual audiograms did not have a negative impact on the outcomes of the RS algorithms, with four out of the six highest ASR scores being associated with individual audiograms. This effect cannot be explained by better PTAs in individual audiograms, which are generally worse than the mean audiograms reported by Humes (2021, see right panel of Figure 5).

**Figure 9:**
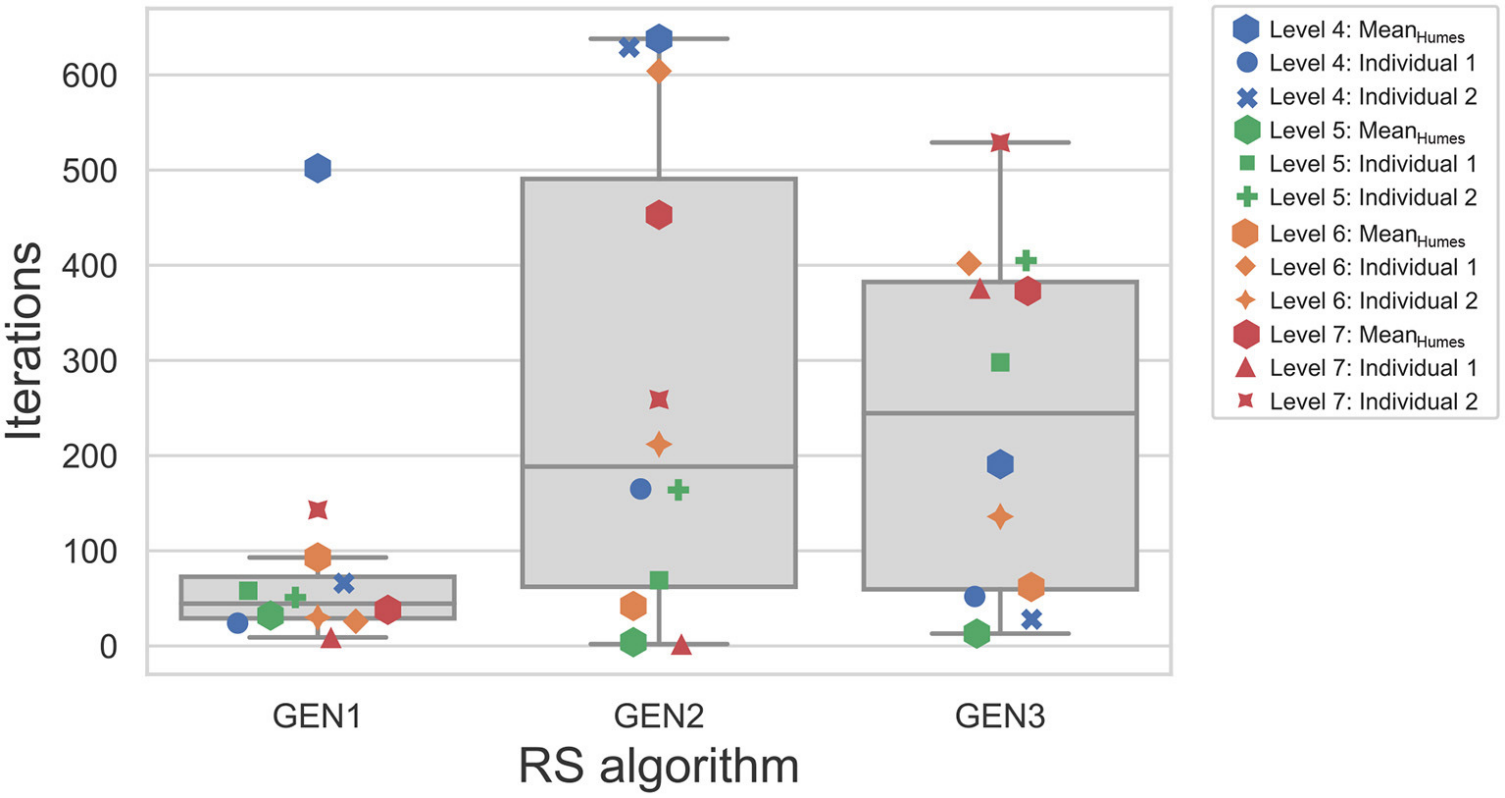
Distribution of the number of iterations needed to reach the highest ASR score that was reached by the three RS algorithms. Otherwise as in Figure 6.

As ASR scores varied as a function of the input audiogram and the RS algorithm, the highest ASR score achieved by all three algorithms (ASR_*Common*_) for the same audiogram was used to compare their speed of convergence. Figure 9 shows the median and individual number of iterations needed by each algorithm to reach ASR_*Common*_ for each of the 12 audiograms. ASR_*Common*_ is sometimes achieved very early during the RS: for GEN1, GEN2, and GEN3, the minimum number of iterations that were needed to achieve ASR_*Common*_ are 9, 2, and 13, respectively. GEN1 is generally faster and reaches, for ten out of the 12 audiograms, ASR_*Common*_ in less than 100 iterations. An outlier is however observed for the mean audiogram corresponding to WARHICS level 4, for which 504 iterations are used by GEN1. The convergence speed for GEN2 and GEN3 is more broadly distributed than for GEN1. A linear mixed model, with the type of RS algorithm as a fixed effect and the audiogram as a random effect, was used to assess the significance of these differences. The results indicate that the type of RS algorithm has a significant effect on convergence speed [*F*(2, 22) = 3.6;*p* = 0.043], and corrected *post-hoc* tests confirm that significant differences exist between GEN1 and GEN2 [*t*(22) = −2.5;*p* = 0.039] and between GEN1 and GEN3 [*t*(22) = −2.1;*p* = 0.049].

To assess the existence of an association between convergence speed and severity of HL (as measured by the PTA), Spearman correlations were computed. For GEN1 and GEN2, this relationship is not statistically significant (both ρ≥−0.16, both *p*≥0.63). In contrast, a significant positive correlation is observed for GEN3 (ρ = 0.65;*p* = 0.022). The convergence speed does not seem to depend on the type of audiogram, as fast and slow convergence speeds are observed for both mean and individual audiograms.

### 3.4. Reproducibility of OPRA-RS Outcomes

The reproducibility of OPRA-RS outcomes was assessed in terms of ASR scores, as well as IGs and CTs, by comparing the outcomes of the two repetitions for each RS algorithm. ASR scores obtained after the second repetition of the RS algorithms (data not shown) are again very high, with median scores equal to those obtained during the first repetition: 98% for GEN1, and 96% for GEN2 and GEN3. Wilcoxon tests showed that there was no significant difference between ASR scores across repetitions for any of the RS algorithms (for GEN1: *Z* = −1.3;*p* = 0.18; for GEN2: *Z* = 0.00;*p* = 1; for GEN3: *Z* = −0.22;*p* = 0.83).

Table 1 shows the Pearson correlation coefficients computed for IG functions outputted by the three RS algorithms after each repetition. For a given RS algorithm, each repetition yielded 12 IG functions (one for each audiogram), each composed of 11 frequency-specific IGs. Twelve correlations were thus computed for each RS algorithm, for a total of 36 correlations. Across RS algorithms, correlation coefficients range from 0.84 to 0.99 and are all highly significant (all *p* ≤ 0.002). Average correlation coefficients are near perfect, ranging from 0.95 (GEN2) to 0.97 (GEN1 and GEN3), indicating that, for each RS algorithm, very similar IG functions were found from one repetition to the next. This consistency builds up gradually, with the correlation coefficient between the best IG function selected at the same point of the RS process for the two repetitions increasing as a function of iteration number (see Supplementary Figure),

**Table 1:** Pearson correlations for IG functions yielded by the two repetitions of each RS algorithm.

	**GEN1**	**GEN2**	**GEN3**
*r*_*min*_	0.89	0.84	0.90
*r*_*max*_	0.99	0.99	0.99
*r*_*mean*_	0.97	0.95	0.97

*For each algorithm, 12 correlations were computed (one correlation per audiogram). Minimum (r_min_), maximum (r_max_), and average (r_mean_) correlation coefficients are reported*.

**Figure 10:**
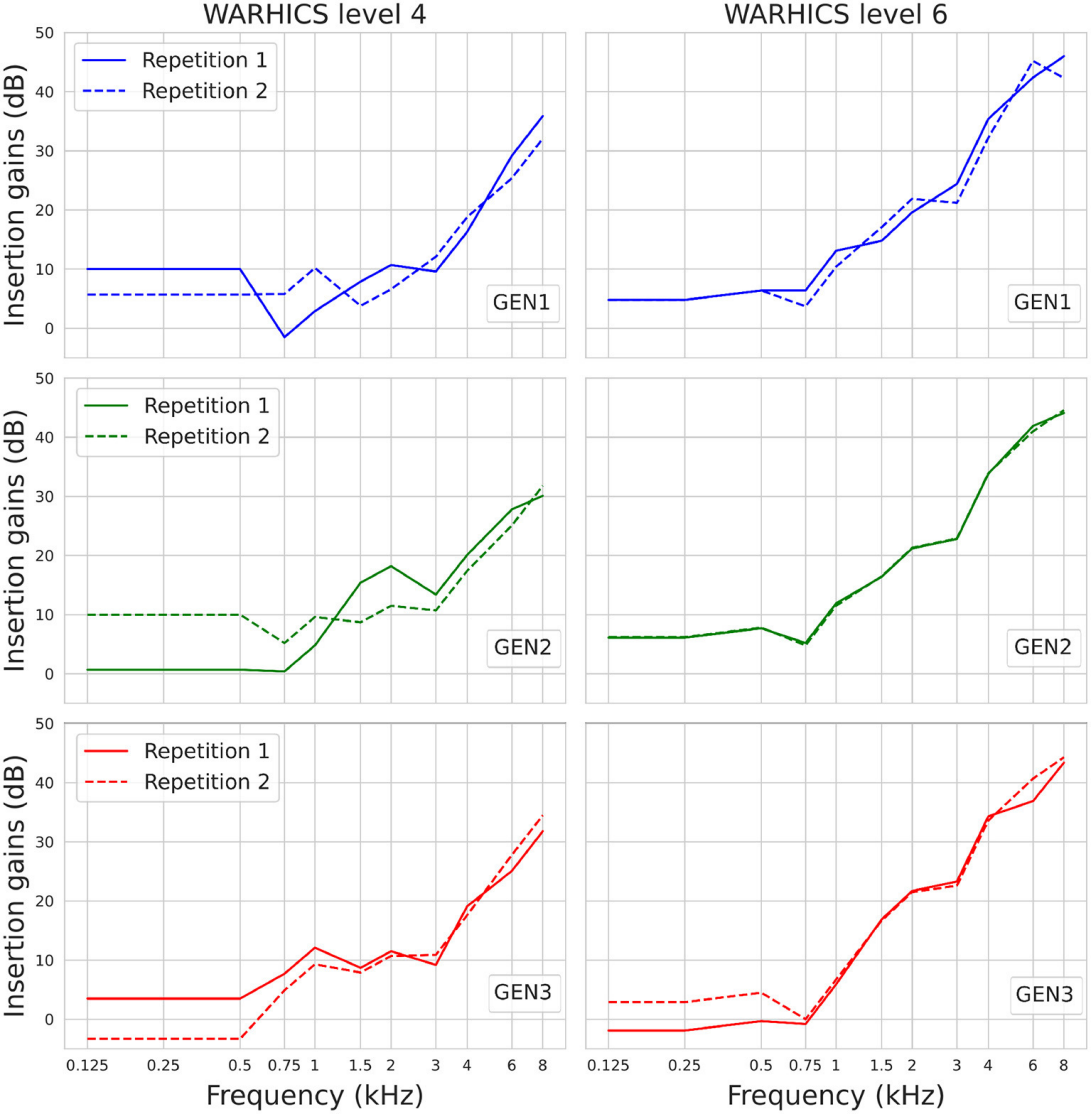
IGs prescribed by OPRA-RS implementing each of the three RS algorithms (see rows) for Humes (2021)'s average audiograms corresponding to WARHICS levels 4 and 6 (see columns).

Figure 10 shows the IGs prescribed by the three RS algorithms for Humes (2021)'s average audiograms corresponding to WARHICS levels 4 and 6. Results are reported for these audiograms as they correspond to the audiograms that, respectively, yielded the weakest and strongest correlation between IG functions found in each of the two repetitions.

To assess to which extent the optimal IG functions found by each RS algorithm differ from the ones found by the other two RS algorithms, Pearson correlations were computed between the IG functions yielded by the different RS algorithms for each audiogram. Twelve correlations were computed for each pair of algorithms (i.e., one correlation per audiogram). Minimum, maximum, and mean correlation coefficients are shown in Table 2. All correlation coefficients are highly significant (all *p* ≤ 0.002), and range from 0.82 to 0.99.

**Table 2:** Pearson correlation coefficients for IG functions obtained by the three RS algorithms for the 12 audiograms.

	**GEN2**	**GEN3**
**GEN1**	*r*_*min*_ = 0.82	*r*_*min*_ = 0.84
	*r*_*max*_ = 0.99	*r*_*max*_ = 0.99
	*r*_*mean*_ = 0.95	*r*_*mean*_ = 0.95
**GEN2**		*r*_*min*_ = 0.89
		*r*_*max*_ = 0.99
		*r*_*mean*_ = 0.96

*For each pair of algorithms, minimum (r_min_), maximum (r_max_), and average (r_mean_) correlation coefficients are reported*.

**Figure 11:**
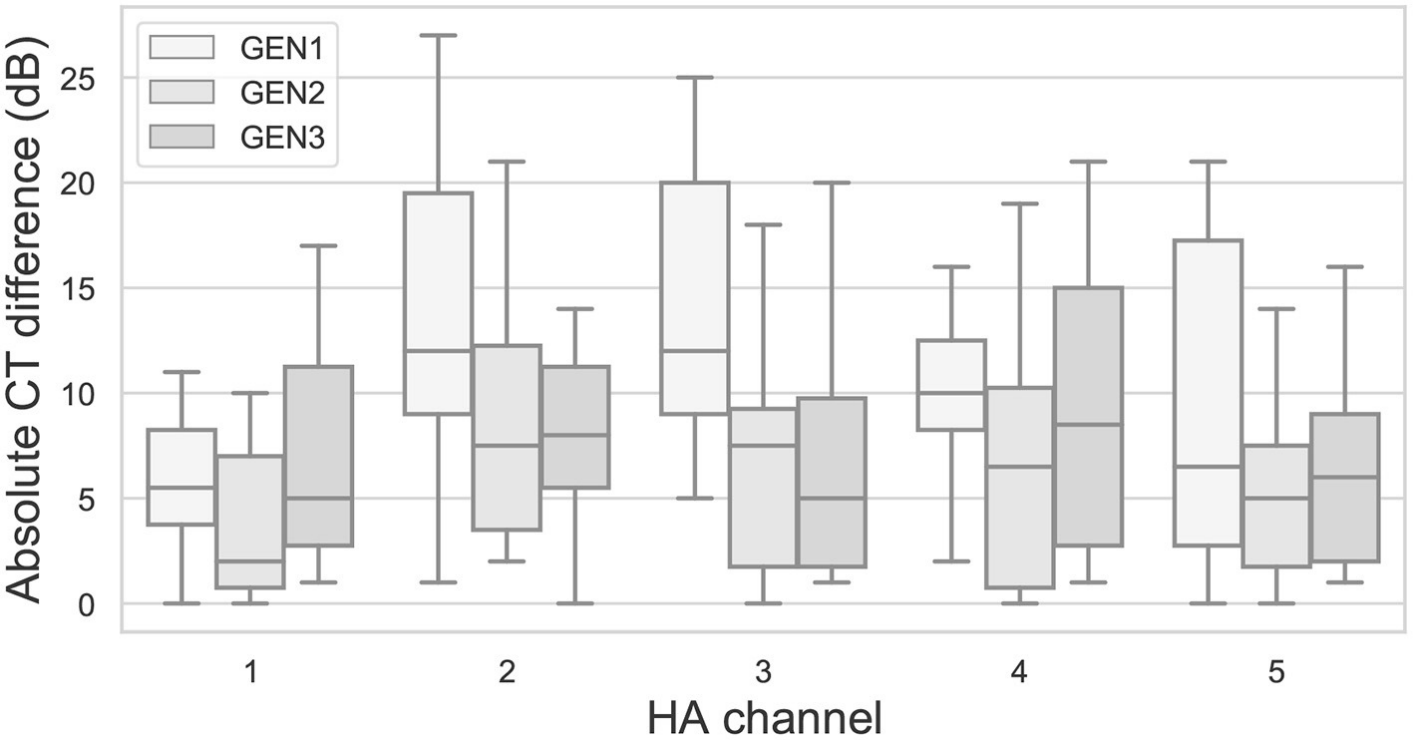
Absolute differences in CTs between repetitions for the three RS algorithms as a function of HA channel. Otherwise as in Figure 6.

Figure 11 shows, for each RS algorithm, the absolute differences in CTs between the two repetitions as a function of HA channel. Across channels, median differences range from 2 to 12 dB, with a maximum individual difference of 27 dB.

## 4. Discussion and Conclusion

This study extends the previous work of Fontan et al. (2020b), in which only the IGSP65s recommended by CAM2 were varied in a very limited range of possible values and in four frequency bands to maximize ASR scores for different audiometric profiles. Here, the use of RS algorithms allowed to vary more parameters (namely, IGSP65s, IGSP85s, and CTs) in more (i.e., five) frequency bands and to use a broader range of possible IGs.

For the three RS algorithms that were used, the ASR scores yielded by optimized IGs were significantly higher than those obtained with the IGs recommended by CAM2. However, the observed differences were small, corresponding to the recognition of one (GEN3) to five (GEN1 and GEN2) out of the 50 words used in the study. It is possible that this small effect size is partly due to a ceiling effect. The observed benefits were indeed larger for the most severe HLs, for which CAM2 yielded lower ASR scores, therefore leaving more room for improvement. In future studies, the use of a larger set of speech stimuli, as well as of an ASR system with a larger lexicon (making the ASR system more prone to confusions), should be explored in order to avoid such ceiling effects.

The IGSP65s prescribed by CAM2 and OPRA-RS were very similar, most likely due to the SII-based equalization. The fact that, despite this similarity, OPRA-RS yielded higher ASR scores than CAM2 might indicate that the other parameters tuned by the RS algorithms (i.e., CTs and IGSP85s) were also determinant for the maximization of ASR scores.

The analysis of the association between PTA and ASR performances revealed that the ASR scores yielded by CAM2 were negatively impacted by HL severity. This might be explained by CAM2 prescriptions not being designed to restore fully audibility for severe cases of HL (Moore et al., 2010b). The fact that, when using OPRA-RS IGs, very high ASR scores were obtained even for the most severe HLs might be related to the fact that OPRA-RS used lower CRs than those recommended by CAM2 in such cases. An additional analysis conducted on OPRA-RS and CAM2 IGs confirms that OPRA-RS CRs were lower (by 1.7 points on average) than those defined by CAM2 for the audiograms corresponding to the most severe HLs used in the study (i.e., audiograms corresponding to the WARHICS level 7). Future research would therefore be warranted to check if OPRA-RS IGs do improve speech intelligibility for actual listeners with severe HLs (in which case the CRs recommended by CAM2 might be regarded as too high) or not (in which case one could put into question the representativeness, for the most severe audiograms, of the ARHL simulation used by OPRA-RS).

As, when simulating severe HLs, the ASR performance can be very low (Fontan et al., 2020a), it was hypothesized that the RS algorithms would need more iterations, and therefore maybe yield lower ASR scores in such cases. The results showed that only the convergence speed of GEN3 was affected by the severity of the simulated HL. As GEN3 only used the IGs prescribed by CAM2 during the first 250 iterations, it is possible that the ASR scores yielded by GEN3 remained very low at this stage, which would explain a slower convergence rate that with GEN1 and GEN2. Contrary to CAM2, the ASR scores yielded by all three RS algorithms were not affected by the HL severity.

Another hypothesis was that the convergence speed would be slower for individual audiograms, whose shape is more erratic than that of the mean audiograms, and that the ASR scores would maybe be lower too for this type of audiograms. This was not the case for any of the three RS algorithms.

GEN1 outperformed the two other RS algorithms in terms of ASR scores and convergence speed, and thus might be the best candidate for future investigations. It is possible that its performances could be further improved if after each iteration the best HA configuration identified across threads would be used as a baseline for the next iteration by all threads, instead of using independent threads.

Finally, the comparison of the outcomes of the RS algorithms across repetitions showed that ASR performance and optimized IGSP65s were reproducible. Some variability was however observed across the IGSP65s selected by the different algorithms, especially at lower frequencies (≤ 0.5 kHz). The fact that, as already observed by Fontan et al. (2020b), ASR scores were impacted by variations in low-frequency IGs, should be interpreted with caution. Indeed, the ability of real HAs to apply IGs in frequencies below 0.2 kHz is very limited, even when wearing ear molds, due to several reasons related to transducer coupling, background noise intrusion, and resulting upward spread of masking (Moore, 2008). It is thus possible that benefits in ASR scores induced by higher gains in lower frequencies would not translate into “real-life” situations. Contrary to the IGSP65s, the CTs showed a high variability across repetitions of the RS algorithms. This may indicate that in the present study the ASR scores were more impacted by IGs and CRs than by CTs, probably because a single presentation level of 65 dB SPL was used.

Taken together, the results obtained in the present study are encouraging and open the way to the use of ASR, combined with RS, to assess very large numbers of possible HA configurations, and to identify the settings yielding maximal speech intelligibility for specific individual audiometric profiles. In the previous study of Fontan et al. (2020b), a similar but simpler optimization chain was used and the optimized settings (which yielded improvements in ASR scores comparable to those yielded by GEN1 and GEN2) led to significant improvements of speech intelligibility and perceived quality in actual older HI persons. The improvements due to this new optimization chain, varying more parameters using smaller setpsizes, might thus be even higher. Moreover, as RS algorithms allow to test very large numbers of conditions, the present study could be extended in several regards. First, only one speech presentation level was used here. Future research should investigate the possibility to use OPRA-RS with speech presented at different levels (e.g., from 50 to 85 dB SPL); this would allow the use of OPRA-RS in real-life scenarios. The speech material should be diversified in order to be more representative of realistic situations (e.g., including speakers of different genders and ages, and different background noises). Finally, other HA parameters such as compression speed, that also impact speech intelligibility, could be investigated using OPRA-RS (see for example Fontan et al., submitted).

## Data Availability Statement

The datasets generated and analyzed for this study can be obtained from the corresponding authors for any research purpose.

## Author Contributions

LF initiated the idea. LG designed and implemented the random-search algorithms and all modifications applied to the previous OPRA prescription chain, under the supervision of JP. MS provided scientific advice about fitting algorithms, and the hearing-aid and hearing-loss simulations. LG, LF, MS, and CF analyzed and interpreted the data. LG, LF, and CF wrote the manuscript. All authors approved the final version of the manuscript.

## Conflict of Interest

This study is part of the development of a future product/service by Archean LABS intended for hearing-aid audiologists. CF acted as a scientific consultant. The remaining authors declare that the research was conducted in the absence of any commercial or financial relationships that could be construed as a potential conflict of interests.

## Publisher's Note

All claims expressed in this article are solely those of the authors and do not necessarily represent those of their affiliated organizations, or those of the publisher, the editors and the reviewers. Any product that may be evaluated in this article, or claim that may be made by its manufacturer, is not guaranteed or endorsed by the publisher.
