# Erratum: OPRA-RS: A hearing-aid fitting method based on automatic speech recognition and random search

**DOI:** 10.3389/fnins.2022.1095750

**Published:** 2022-12-09

**Authors:** Frontiers Production Office

**Affiliations:** Frontiers Media SA, Lausanne, Switzerland

**Keywords:** random search (RS), automatic speech recognition (ASR), hearing aids (HAs), prescription rule, age-related hearing loss (ARHL), insertion gains, compression thresholds

Due to a production error, Figure 1 was wrong in the PDF. Correct Figure 1 appears below.

**Figure 1:**
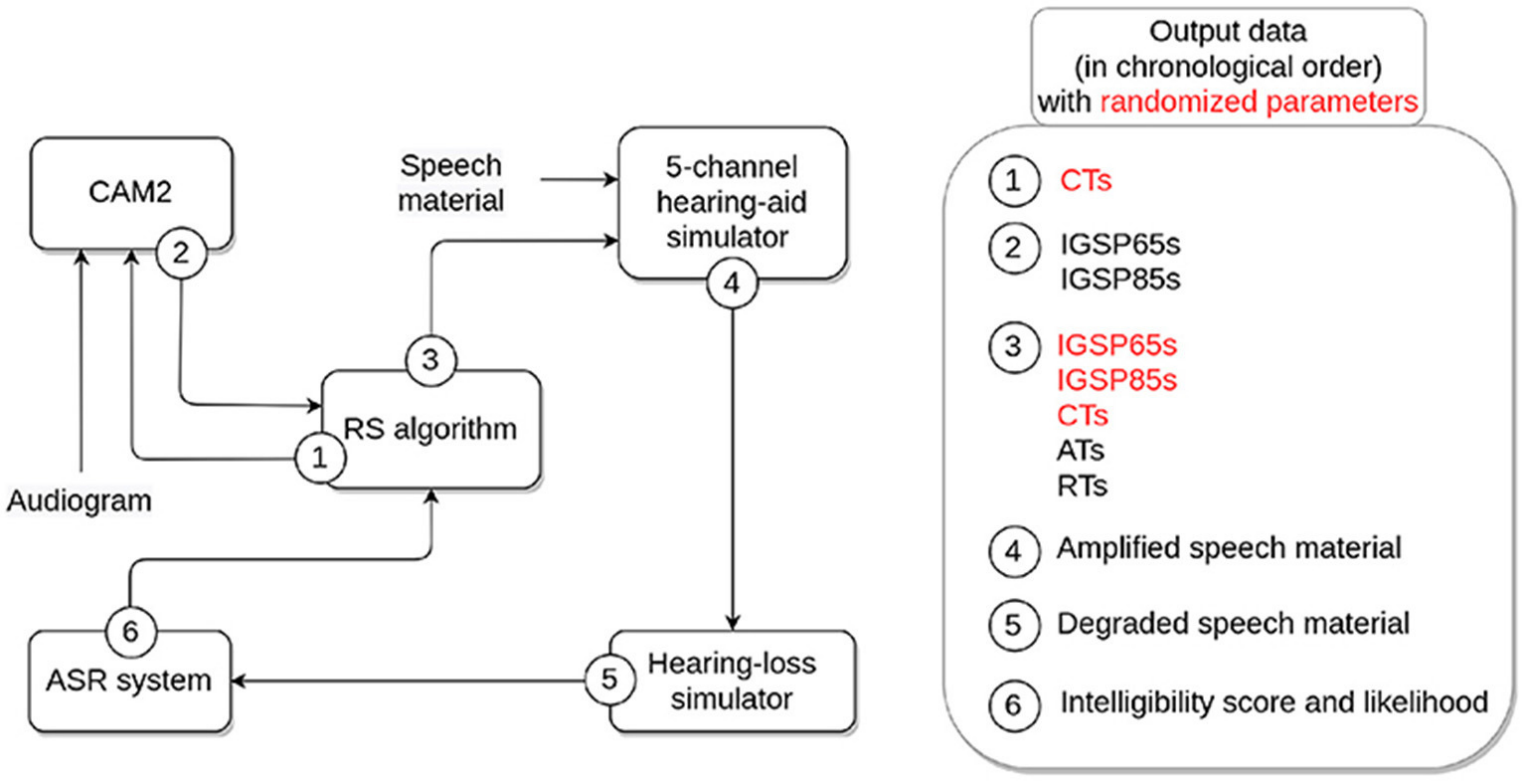
Overview of the components of the OPRA-RS processing chain and associated output data. HA parameters randomized by the RS algorithm appear in red.

The publisher apologizes for this mistake. The original article has been updated.

