# Supplementary material for: OPRA-RS: A Hearing-Aid Fitting Method Based on Automatic Speech Recognition and Random Search
Source: Front Neurosci. 2022 Feb 21;16:779048. doi: 10.3389/fnins.2022.779048 (PMC8899657; doi:10.3389/fnins.2022.779048)
Supplement: Supplementary file 1 [file Image_1.pdf]

## SUPPLEMENTARY FIGURE

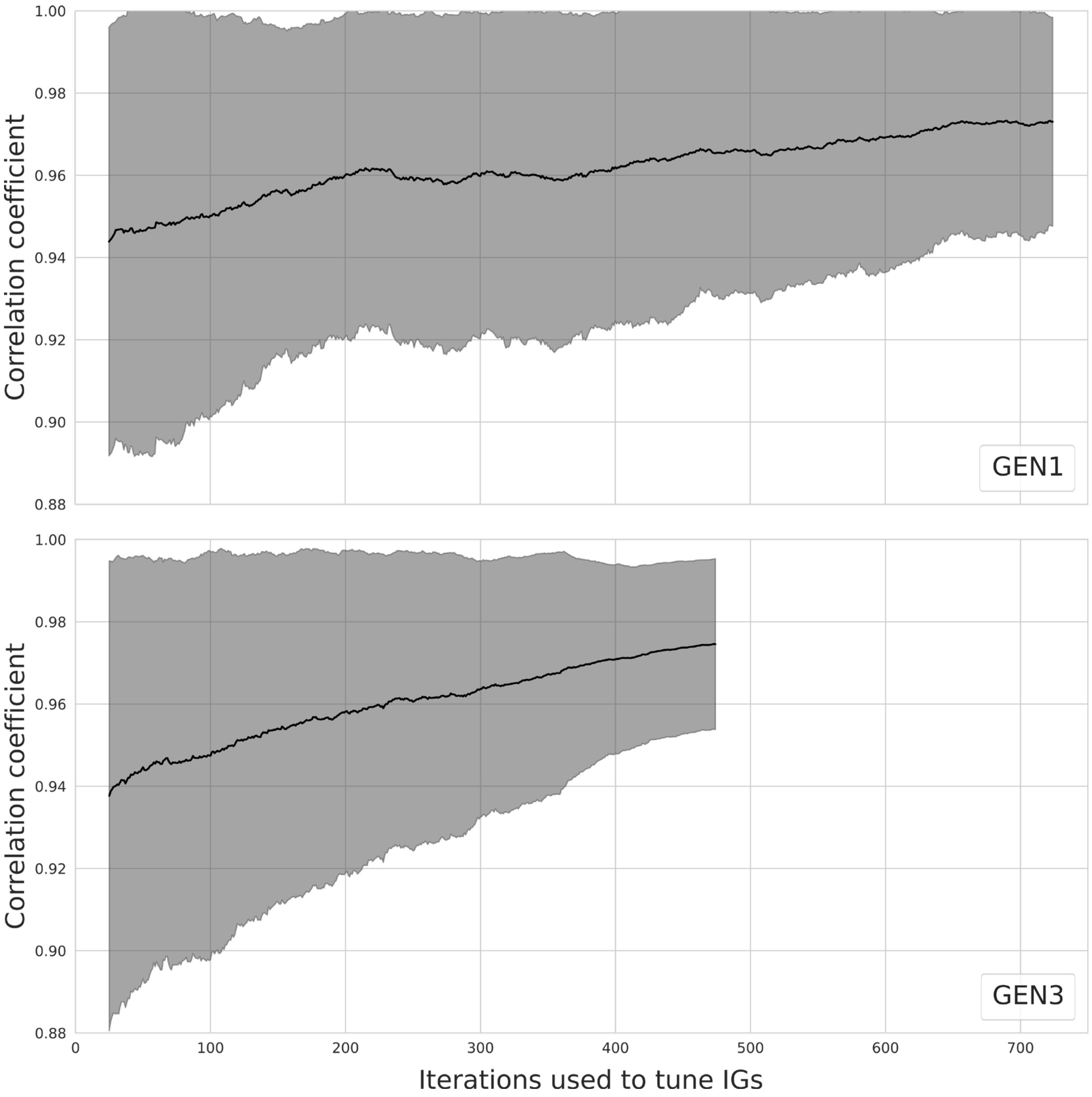


**Supplementary Figure 1.** Pearson correlation coefficients for IG functions found during the two repetitions of GEN1 (top panel) and GEN3 (bottom panel) averaged across the 12 audiograms, as a function of iteration number. The gray area represents $\pm 1$ SD. For each iteration $i$, the average correlation coefficient and SD are calculatedfor iterations $i - 25$ to $i + 25$. Only iterations used to tune IGs are shown.
